# Land cover and NDVI are important predictors in habitat selection along migration for the Golden-crowned Sparrow, a temperate-zone migrating songbird

**DOI:** 10.1186/s40462-022-00353-2

**Published:** 2023-01-13

**Authors:** Autumn R. Iverson, Diana L. Humple, Renée L. Cormier, Josh Hull

**Affiliations:** 1 Department of Animal Science, University of California, Davis, One Shields Ave, Davis, CA 95616 USA; 2 Point Blue Conservation Science, 3820 Cypress Drive #11, Petaluma, CA 94954 USA

**Keywords:** Bird, Golden-crowned Sparrow, GPS, Habitat selection, Migration, Stopover

## Abstract

**Background:**

Migrating passerines in North America have shown sharp declines. Understanding habitat selection and threats along migration paths are critical research needs, but details about migrations have been limited due to the difficulty of tracking small birds. Recent technological advances of tiny GPS-tags provide new opportunities to delineate fine-scale movements in small passerines during a life stage that has previously been inherently difficult to study.

**Methods:**

We investigated habitat selection along migration routes for a temperate-zone migratory passerine, the Golden-crowned Sparrow (*Zonotrichia atricapilla*), given GPS tags on California wintering grounds. We used a resource selection function combined with conditional logistic regression to compare matched sets of known stopover locations and available but unused locations to determine how land cover class, vegetation greenness and climate variables influence habitat selection during migration. We also provide general migration descriptions for this understudied species including migration distance, duration, and elevation, and repeated use of stopover areas.

**Results:**

We acquired 22 tracks across 19 individuals, with a total of 541 valid spring and fall migration locations. Birds traveled to breeding grounds in Alaska and British Columbia along coastal routes, selecting for shrubland and higher vegetation greenness in both migration seasons as well as grasslands during fall migration. However, model interactions showed they selected sites with lower levels of greenness when in forest (both seasons) and shrubland (fall only), which may reflect their preference for more open habitats or represent a trade-off in selection between habitat type and productivity. Birds also selected for locations with higher daily maximum temperature during spring migration. Routes during spring migration were lower in elevation on average, shorter in duration, and had fewer long stopovers than in fall migration. For two birds, we found repeated use of the same stopover areas in spring and fall migration.

**Conclusions:**

Using miniaturized GPS, this study provides new insight into habitat selection along migration routes for a common temperate-zone migrating songbird, contributing to a better understanding of full annual cycle models, and informing conservation efforts. Golden-crowned Sparrows selected for specific habitats along migration routes, and we found previously unknown behaviors such as repeated use of the same stopover areas by individuals across different migratory seasons.

## Background

Seventy percent of temperate-zone migrating passerines have shown widespread decline in western North America over the last few decades, likely due to a combination of rapid rates of landcover and climate change [1]. Of the 3.2 billion birds lost in North America over the past 50 years specifically, native sparrows make up about a quarter of that loss (~ 800 million individuals) and have seen a 35% decline in numbers since 1970 [2].

It is increasingly recognized that in order to fully understand what limits populations of migratory bird species, it is important to consider the complete annual cycle [3]. Along migration routes, birds can face many threats such as increased nutritional needs, vulnerability to novel predators, extreme weather conditions, and navigational challenges. Adding to these inherent risks are anthropogenic changes across landscapes, such as habitat loss and climate change. For passerines, there are indications that migration is where most annual mortality occurs [4], yet most research on migratory birds has occurred during the breeding season, with a lesser focus on winter and migration. Migration is the least understood life stage and delineating habitat quality and threats along migration paths are critical research needs [5].

Some species undergo their migrations with long flights interspersed with major stopover periods, such as many shorebirds that fly thousands of kilometers non-stop [6]. This has been referred to as long-bout migration, while species that migrate in shorter bursts with more frequent stopovers are referred to as short-bout migrants [7, 8]. This short-bout migration strategy is thought to be common for landbird migrants in the northern hemisphere [7], with birds refueling during the day and migrating in the first few hours of the night [8, 9]. Many passerine species are seasonal migrants that travel between wintering and breeding grounds, in general breeding in higher latitudes and migrating to lower latitudes for the winter [10]. While migratory routes are relatively understudied for passerines, it is known that conditions experienced during migration can have carry-over effects on population dynamics at breeding and wintering grounds. For example, a bird’s body condition upon arrival at breeding grounds is in part a reflection of the quality and quantity of resources encountered at stopover sites on migration, and can influence breeding success [11].

The Golden-crowned Sparrow (*Zonotrichia atricapilla*) is a short-bout temperate-zone North American migrant that winters from southern British Columbia to northern Baja California and breeds in parts of Alaska, the Yukon, and British Columbia. They are considered a medium-distance migrant, with the entire population migrating seasonally between these locations [12]. Golden-crowned Sparrows are found in a variety of habitats during breeding and wintering including shrublands, riparian thickets, scattered conifers near or above tree line, gardens, and urban areas [12]. Previous research on the migration of Golden-crowned Sparrows has shown strong regional migratory connectivity [13], although individuals tracked from the same wintering grounds did not travel to the exact same breeding grounds [13–15]. The structure of song dialects on breeding and wintering grounds indicate that Golden-crowned Sparrows may exhibit chain migration where the birds that winter at the highest latitudes also breed at the highest latitudes [16, 17]. Despite these recent studies, knowledge of their habitat selection along migration routes remains limited.

Recent technological advances have led to increasingly accurate GPS (Global Positioning System)-tracking devices small enough for some passerines to carry during migration, providing new opportunities to delineate fine-scale movements in this and similar species during a life stage that has previously been inherently difficult to study. In this study, we deployed GPS tags on Golden-crowned Sparrows wintering in California to describe their migration behavior at stopover locations along their route in more detail than possible in previous studies. Specifically, we sought to investigate how habitat and climate affect the choice of stopover sites. Based on known Golden-crowned Sparrow habitats at breeding and wintering locations, we expected they would use similar habitats at stopover locations (e.g., shrublands and other more open habitat types) and generally avoid land cover with dense canopy vegetation such as heavily forested areas. Additionally, the seasonality of productivity across landscapes is expected to be an important migratory cue as it would represent food availability, and satellite measures on North American ecological productivity show this productivity influences bird migration strategies [18]. Therefore, we tested how a measure of vegetation greenness (Normalized Difference Vegetation Index; NDVI) was related to stopover-site choice on migration. NDVI values have been shown to be correlated with net primary productivity [19] and since birds need to refuel at stopover locations, we expected Golden-crowned Sparrows to stopover in areas with higher levels of NDVI. Finally, we contribute to the general knowledge of Golden-crowned Sparrow migration by describing migration distances and durations as well as how long birds stayed at stopovers sites on their way between wintering and breeding locations. This information can contribute to full annual cycle models for this species. Also, knowing habitat preferences for a species can help us understand how they may adapt, or struggle to adapt, to future habitat changes from development, wildfires and climate change.

## Methods

### Capture and processing

**Fig. 1:**
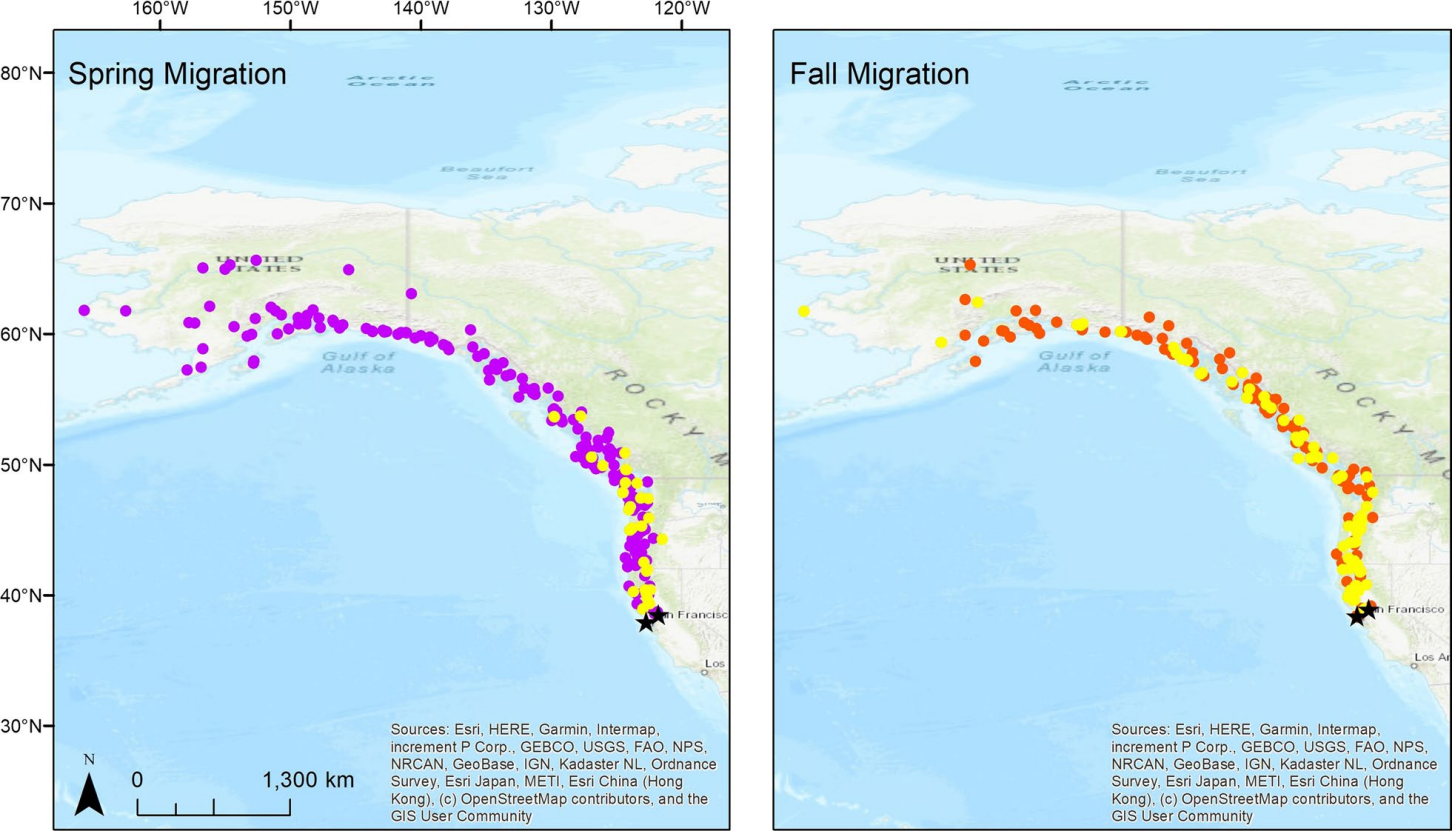
Filtered GPS locations for migrating Golden-crowned Sparrows tagged at wintering sites in California 2019–2020. Spring migration locations are shown in the left panel (n = 246) and fall migration in the right panel (n = 295). Wintering sites in northern California are indicated by black stars. Purple (spring) and orange (fall) represent locations with shorter stopovers, and yellow locations represent areas with longer stopovers (at least 2 locations, i.e., >  = 3 days, within 10 km of each other for an individual bird)

We captured birds at two study sites in northern California: (1) around the Point Blue Conservation Science office and residence at the historic Hagmaier Ranch in Olema Valley, Point Reyes National Seashore, Marin County (37.971, -122.731), in the San Francisco Bay Area; and (2) around the Zoology Field Building on the University of California, Davis (UC Davis) campus, Yolo County, in the Central Valley (38.529, -121.783; Fig. 1). The Point Reyes site was surrounded by miles of natural habitat with no large urban setting nearby, and the small towns of Olema and Bolinas each approximately eight kilometers away. The immediate surroundings included a mix of riparian (dominated by red alder, *Alnus rubra*) and coastal scrub (including coyote brush, *Baccharis pilularis* and blackberry, *Rubus* sp.) habitats. The UC Davis campus site was within a mix of urban and agricultural areas in the town of Davis, near a riparian corridor and open woodland, with native valley oak (*Quercus lobata*) and California black walnut (*Juglans hindsii*), and non-native tree of heaven (*Ailanthus altissima*) and *Eucalyptus*.

We captured Golden-crowned Sparrows for tagging in winters 2018–2019 and 2019–2020, then recaptured them the following fall/winter after migration to and from their northern breeding grounds (Table 1). We primarily used walk-in traps baited with seed (millet or mixed seeds) and passive mist nets. We recorded morphometric and demographic data, including weight, wing chord length, fat score (on a scale from 0 to 7), and age. To determine eligibility for a tag overall and for specific tags (tags varied slightly in weight), we then calculated lean body weight by subtracting a coarse estimated weight of fat, as adapted from lean weight guidelines for White-crowned Sparrows (*Zonotrichia leucophrys* [20]*;*) modified for use by Point Blue Conservation Science. If there was no fat present (fat score = 0), we did not subtract any amount from the weight (lean weight = weight); if fat was light (present in a trace amount up to half-filling the furculum and the abdomen showing small patches; fat scores 1–3) we subtracted 1.5 g; if fat was medium (present in the furculum from 2/3 filled to level with clavicles, and the abdomen had a covering pad fat score = 4), we subtracted 4.0 g; and if fat was heavy (the furculum was bulging with fat; fat scores 5–7) we subtracted 8.0 g. We determined age by plumage [20], especially the crown plumage variation for which we followed a color guide [21]. Although we did not use age or sex in our analyses, we provide this information for transparency on the sample of birds involved in this study (Table 1).

**Table 1:** Details of individual Golden-crowned Sparrow tag deployments at wintering sites in northern California, 2019–2020

Tag number	Tagging date	Age	Sex	Mass	Fat score	Lean weight	Tag weight	Tag + harness weight	% of body weight	Tag status
*Point Reyes*										
49216	1/4/2019	AHY	U	34.1	3	32.6	1.0	1.2	3.7	R
49209	1/4/2019	SY	F	31.8	3	30.3	1.1	1.3	4.3	R
49218	1/4/2019	SY	F	36.1	3	34.6	1.1	1.2	3.5	R
49198	1/4/2019	AHY	U	37.6	4	33.6	1.1	1.3*	3.9	TL
49222	1/4/2019	SY	F	33.5	4	29.5	1.1	1.2	4.1	R
49214	1/11/2019	AHY	U	34.9	2	33.4	1.0	1.3	3.9	R
49199	1/11/2019	AHY	U	37.4	3	35.9	1.0	1.2	3.3	NR
49200	1/11/2019	SY	F	34.1	3	32.6	1.0	1.2	3.7	R
49204	1/19/2019	AHY	U	31.4	2	29.9	1.0	1.3	4.3	NR
49220	1/19/2019	SY	U	32.3	2	30.8	1.0	1.2	3.9	TL
49207	1/19/2019	ASY	U	29.6	2	28.1	1.0	1.3	4.6	NR
49208	1/19/2019	AHY	U	35.1	3	33.6	1.0	1.3	3.9	R
49211	1/19/2019	AHY	U	31	3	29.5	1.0	1.2	4.1	NR
49215	1/19/2019	AHY	U	32.2	3	30.7	1.0	1.3*	4.2	TL
49221	1/19/2019	ASY	U	32	3	30.5	1.0	1.3	4.3	NR
49190	3/29/2019	AHY	U	32.2	2	30.7	1.1	1.2	3.9	NR
49201	3/29/2019	AHY	F	29.3	2	27.8	1.0	1.2	4.3	R
*ZFL*										
49212	2/19/2019	ASY	U	31.8	3	30.3	1.1	1.3	4.3	NR
49206	2/19/2019	AHY	F	32.1	2	30.6	1.0	1.2	3.9	R
49196	2/19/2019	ASY	U	34.8	2	33.3	1.1	1.2	3.6	R
49223	2/19/2019	ASY	U	38.3	3	36.8	1.0	1.2	3.3	*TL*
49210	2/20/2019	ASY	U	36.4	3	34.9	1.0	1.2	3.4	NR
49189-a	2/20/2019	ASY	F	32.1	2	30.6	1.0	1.2	3.9	R
49202-b	2/20/2019	ASY	M	36	3	34.5	1.0	1.3	3.8	R
49219	2/21/2019	AHY	U	31.8	2	30.3	1.0	1.2	4.0	NR
49193	2/21/2019	ASY	U	35.8	2	34.3	1.0	1.2	3.5	NR
49213	2/21/2019	AHY	U	35	3	33.5	1.1	1.2	3.6	NR
49191	2/21/2019	AHY	M	34.1	0	34.1	1.0	1.2	3.5	R
49192-c	2/21/2019	SY	M	35.2	3	33.7	1.0	1.2	3.6	R
49197	2/24/2019	AHY	U	32.8	3	31.3	1.0	1.2	3.8	NR
49205-d	2/24/2019	AHY	M	36.2	2	34.7	1.1	1.3	3.7	R
49217-e	2/24/2019	AHY	M	38.5	3	37	1.0	1.2	3.2	R
49203	3/1/2019	AHY	U	34.8	3	33.3	1.1	1.2	3.6	NR
49194-f	3/1/2019	AHY	M	32.4	2	30.9	1.0	1.2	3.9	R
49195-g	3/1/2019	AHY	M	33.6	2	32.1	1.0	1.3	4.0	R
49772	2/4/2020	SY	U	33.9	2	32.4	1.1	1.2	3.7	NR
49779	2/4/2020	ASY	U	35.1	2	33.6	1.1	1.1	3.3	NR
49769-e	2/4/2020	AHY	U	N/A	3	37**	1.1	N/A	3.2	NR
49774	2/4/2020	ASY	U	28.7	3	27.2	1.0	1.2	4.4	NR
49780	2/4/2020	SY	M	31.4	0	31.4	1.1	1.2	3.8	R
49770-g	2/4/2020	AHY	M	N/A	2	32.1**	1.1	1.2	3.7	R
49771	2/4/2020	SY	F	29.7	2	28.2	1.0	1.3	4.6	R
49870	2/4/2020	ASY	M	36.7	2	35.2	1.2	1.3	3.7	R
49778-b	2/4/2020	ASY	M	35.4	2	33.9	1.1	1.2	3.5	R
49777	2/4/2020	AHY	M	34.4	3	32.9	1.1	1.3	4.0	R
49869-c	2/6/2020	AHY	M	35.6	2	34.1	1.2	1.3	3.8	NR
49871-f	2/6/2020	ASY	U	34.6	2	33.1	1.2	1.2	3.6	NR
49773-d	2/6/2020	ASY	M	35.1	3	33.6	1.1	1.2	3.6	NR
49775	2/6/2020	SY	F	29.8	2	28.3	1.0	1.2	4.2	R
49776-a	2/6/2020	ASY	F	33.4	3	31.9	1.1	1.2	3.8	R
			Mean	33.8	2.5	32.3	1.1	1.2	3.8	
			SD	2.4	0.8	2.4	0.1	0.1	0.3	

Tag numbers followed by a letter indicate individual birds that were tagged twice (i.e., tags with “a” were put on the same individual). Birds were aged as After Hatching-Year (AHY), Second Year (SY), or After Second-Year (ASY) based on their calendar year (Pyle 1997), and some were sexed genetically as Female (F) or Male (M), or otherwise remained Unknown (U). Fat scores and lean weight are described in the Methods. The % of body weight is calculated with the tag + harness weight and the lean body weight. All weights/masses are in grams. Tag status: R = tag recovered, TL = tag lost on a retrieved bird (italics indicates bird for which tag was lost within 1 day of attaching it that later became a control), NR = tag not recovered (bird not observed/recaptured in return year)

*Harness + tag weight estimated

**Lean weight calculated from mass and/or fat scores recorded on another capture event

We banded individuals using numbered aluminum bands provided by the US Geological Survey Bird Banding Lab and plastic butt-end color bands to enable identification of unique individuals in the field and enhance recovery efforts. We used the same banding protocol for a subset of non-tagged control birds to assess tag effects on return rates without having to recapture them. The weights of tagged birds (mean: 33.8 g, range 28.7–38.5; Table 1) were similar to control birds (mean: 33.7 g, range: 28.3–42.4 g). We also made one bird a control because it lost its tag shortly after deployment (within 1 day). In addition to the birds we tagged (numbers provided below), in 2019 we banded 16 control birds at Point Reyes and 15 at UC Davis, and in 2020 we banded 15 new control birds at UC Davis. At initial banding of control birds, we collected two tail feathers for stable isotope analysis as part of a related study (Iverson et al. *In Prep*), and 8–10 contour feathers for genetic sexing. For tagged birds, feather collection occurred upon tag retrieval. We released birds at the site of capture.

### Tag deployment

We deployed 50 archival 1.0–1.2 g GPS tags (PinPoint-10 GPS store-on-board tags from Lotek Wireless; weights varied as tags were handmade) with an expected horizontal accuracy within 10 m. This included 17 tags deployed at Point Reyes in 2019, 18 at UC Davis in 2019, and 15 at UC Davis in 2020. For 7 of 10 individuals at the UC Davis site that returned with a tag in 2019, we gave them a replacement tag for another year of tracking (with 8 additional birds receiving tags for the first time in 2020). Tags were attached using leg-loop harnesses [22]. The tag + harness weight was determined during tagging by weighing the tag + harness before attaching to the bird, cutting off the excess Stretch Magic jewelry cord used for the harness after sizing it for the individual bird, weighing those removed pieces, and subtracting from the total weight (and/or it was also assessed upon removal of the tag). If both methods were used to assess tag + harness weight, then the higher of the two values is reported. The combined tag and harness weight was less than 5% of the bird’s lean body weight (range = 3.2–4.6% of the lean body weight, mean 3.8% ± SD 0.4%; Table 1).

As tags needed to be retrieved with remaining battery life in order to download the data, it was recommended by Lotek Wireless to program for approximately 60 locations to ensure battery life lasted throughout a long deployment. Because average spring and fall migration durations for Golden-crowned Sparrows were previously determined using light-level tags to range from 22.8 to 31.9 days and occur from April 19 to May 31 in the spring and September 2 to October 26 in the fall [13], in the first year, we programmed tags to collect a location every other day during these expected spring and fall migrations. With the remaining available points, we programmed tags to collect locations every ninth day during the breeding season (June 2–Aug 31). This resulted in up to 61 GPS fixes anticipated per bird: 22 during the spring migration period, 11 during time on breeding grounds, and 28 during the fall migration period. After evaluating the 2019 data, we adjusted the dates slightly for the 2020 programming to collect a location every other day during revised and expanded expected spring (Apr 13–May 27) and fall (Aug 31–Oct 28) migrations and every thirteenth day during the breeding season (May 29–Aug 28); this still resulted in up to 61 GPS fixes per bird but with 23 locations during spring migration, 8 on breeding grounds, and 30 during fall migration. Each fix was programmed to be taken in the morning, around 9:00 am when the birds were not expected to be actively migrating. Based on previous recapture rates for this species from studies using light-level tags [13, 14], we expected to retrieve about 30–50% of GPS tags, or about five to eight at each location in each year.

### Location filtering

Each estimated GPS location came with an accuracy estimate called the Horizontal Dilution of Precision (HDOP). HDOP values of < 1–5 are considered ideal to good, 5–10 are considered moderate, 10–20 are considered fair, and > 20 are considered poor [23]. Therefore, we filtered out any GPS locations with an HDOP > 20 (n = 5 from spring migration and n = 9 from fall migration) or outside the known migration range (n = 1 location in Idaho on spring migration between the bird being in B.C and Alaska). From these filtered locations, we classified locations as migratory stopovers if they were at least ~ 1–2 km away from the cluster of points representing breeding or wintering areas and outside the time period that the bird was established at those breeding/wintering sites.

### General migration description

We calculated total migration distance by summing the consecutive geodesic distances between filtered migration locations, using the R package geosphere [24]. For the starting and ending locations of migration distance, we used the centroid of home ranges calculated at winter and breeding areas (kernel density estimates or minimum convex polygons, Iverson et al. *In Prep*). As tags did not collect points daily, some stopover locations during migration would have been missed; therefore, our migration distances are likely underestimates. We also extracted elevation data at each point using the elevatr package in R [25] for points in the United States and using the Canada3D digital elevation model (30 arc seconds) produced by the Canadian Forestry Service [26] for points in Canada.

Migration duration was estimated from the date of the first location recorded on migration to the date of the first location recorded at the destination. As tags did not collect points daily, and the switch to/from migration may have happened when tags were less frequently collecting points (e.g., during the breeding settings), we also report the uncertainty in these values. Specifically, we calculated the number of days between 1) the last known date at the area they were leaving (wintering and breeding areas) and the start of known migration and 2) the end of known migration and the first known date at their destination, and summed both values for a total value of the uncertainty in the number of days spent on migration.

To understand how long birds stayed at a stopover during migration, we classified locations within 10 km of each other for each track as the same stopover area, as other passerine species have been shown to make movements at this scale within stopover areas (e.g. ~ 1–21 km for White-throated Sparrows *Z. albicollis*, [27, 28]; up to ~ 10 km for Swainson’s Thrush *Catharus ustulatus* and Hermit Thrush *C. guttatus*, [27]). As points during migration were at least two days apart and taken in the morning, with birds expected to travel at night, two points within 10 km would indicate staying in the same general area for at least three days. We counted how many times these longer stopovers (defined as a minimum of 3 days) occurred for fall and spring migration. We conducted a paired t-test for any tag that recorded stopovers in both spring and fall to determine if the number of long stopovers varied seasonally; we included tags in this analysis as long as they had at least one stopover recorded in each season (i.e. not necessarily recording data for all of fall migration). We also assessed the number and proportion of locations that occurred in different habitats (shrubland, needleleaf forest and grassland) by stopover type in fall and spring to see if certain habitat types were more represented at short versus long stopovers.

### Defining available habitat

We evaluated habitat use by Golden-crowned Sparrows at stopover sites with resource selection functions (RSFs), which use random locations to compare heterogeneity in habitat characteristics to the presence or absence of animal locations [29]. That is, we compared each location (“presence”) to a set of available locations unused by that same individual for the same day and in the same general area (“absences”), and then compared the associated environmental characteristics between these used and unused sites. Generally, the observed and unobserved (random) locations are compared using logistic regressions. However, we used a conditional logistic regression where known stopover locations were matched with a spatially limited set of random locations to ensure that random locations represented true absences [30, 31]. Inference on resource selection is determined by available but unused locations, and so it is critical to appropriately define the available habitat space. Therefore, we added a sensitivity analysis to address how different patch sizes of assumed available habitat might affect our model results. To do this, we created unobserved (random) locations at varying distances from the observed locations.

For the main analysis, we created 20 random points within 50 km of each real location (i.e. across a 100 km diameter circular area around the presence point), since the average distance moved during migration was ~ 100–120 km a day (calculated across all tags). Therefore, assuming birds moved an average 100 km each night to land at the known presence point, the circle for random points would allow 50–150 km of movement from a (theoretical) previous day’s location, a range that encompasses almost all daily movement distances calculated from observed locations. Alternatively, if the bird was taking a longer stopover and had not actively migrated the previous night, the circle would allow for 0–100 km of movement from the previous day’s location, thereby accommodating no movement as well as the average movement assumption of 100 km. Random points in each circle were at least 1 km from each other to accommodate the resolution of environmental variable data. Additionally, we made random points at least 10 km from the observed location to increase likelihood of differentiation between the characteristics of the known stopover and random locations. Random points were only generated on land.

For the sensitivity analysis, we created four other sets of random points in the same way as above, at a maximum distance of 15, 25, 75 and 100 km from each known stopover location (i.e. 30, 50, 100 and 200 km diameter circular areas around each observed location). For each scale, we generated 20 random locations, except for the smallest scale for which we generated 10 random points, using ArcMap 10.8 [32]. When using conditional logistic regression, a small number of locations for comparison in each matched set is not expected to affect parameter estimation, with 10–20 random locations or less commonly used [33–35].

### Environmental variables

Since land cover type and/or the extent of green vegetation (“greenness”) may be important for stopover locations (for refueling or cover), at each location, we extracted the habitat type and an NDVI value of vegetation greenness. Land cover type was determined using the 2015 North America Land Change Monitoring System map (NALCMS; [36]) which had a resolution of 30 m and 19 land cover classes. Based on initial evaluation of the most commonly represented habitat types at known stopover and random points, we created binary variables of presence or absence (0 or 1) of three land cover classes at these points: temperate or sub-polar grassland (hereafter grassland), temperate or sub-polar needleleaf forest (hereafter needleleaf forest), and temperate or sub-polar shrubland (hereafter shrubland).

NDVI (Terra MODIS Vegetation Indices) was downloaded from the USGS AppEEARS website [37] and had a resolution of 1 km and 16 days. As NDVI data was only available every 16 days, known stopover and random locations were given the NDVI value that spatially overlapped and was closest in time. If the location was exactly 8 days from prior and future NDVI values, it was matched with the NDVI value of 8 days prior. Raw NDVI values were scaled by multiplying by 0.0001 to translate the unscaled values in the downloaded data to valid NDVI values between -1 and 1. Lower NDVI values represent areas such as those with barren rock, water, and snow (i.e. a value close to zero represents no greenness). The higher the NDVI value, the denser the green vegetation, with values close to 1 (i.e. around 0.8–0.9) representing the highest possible green leaf density [38].

To assess how climate may influence stopover locations, we downloaded daily precipitation and temperature data from the USGS AppEEARS website [39]. We chose the DAYMET.004 layer which provided daily precipitation (mm), minimum temperature (℃), and maximum temperature (℃) data for both the United States and Canada at a spatial resolution of 1 km.

All variables were standardized prior to analysis and no variable combination showed correlations greater than ± 0.7, thus none were dropped based on this. Random points were given the same date as their matched known stopover location for the purposes of daily environmental data extraction. After extracting the environmental data for each location, we dropped any known locations with missing data. This included 13 known locations in spring and eight in fall that were missing precipitation, temperature, NDVI and/or habitat type data.

### Statistical analysis

We performed conditional logistic regression using the survival package [40] in R [41] to determine the importance of the environmental characteristics at each stopover point. In conditional logistic regression, each case (the presence location) is matched with controls (the random locations), and each match represents a stratum. Conditional logistic regression models have no intercept term and any desired random effects would be included as random regression coefficients [31]. However, individual-specific random slopes are extremely difficult to fit in conditional logistic regressions [42] and we did not have enough strata per individual to estimate this. Therefore, our analysis assumes homogeneity across individuals in site choice.

We performed two separate analyses: one for spring migration locations and one for fall migration. Three birds were successfully tracked twice (across two consecutive years), and as we assumed homogeneity across individuals in site choice, we included both tracks from these birds in analyses; however, we also ran the models with one track removed for each of these birds to ensure their individual choices were not strongly influencing results. We acknowledge there may also be differences among birds by population, age, or sex, but sample sizes were not large enough to examine all these factors and our results represent the overall average.

For each season, our global model included climate variables (precipitation and temperature), habitat type, NDVI, and NDVI/habitat interactions. Grassland was used only for fall models because there were few locations in grassland at known stopover and random locations for spring. Competing models were simplifications of the global model. We compared models using Akaike’s Information Criterion corrected for small sample bias (AIC_c_) using the MuMIn package in R [43]. AIC is an information-theoretical approach that measures the information lost in each model across a given set of candidate models. Models with ΔAIC < 2 suggest substantial support for a model [44]. We averaged any competing models if more than one model had ΔAIC < 4, were within 2 ΔAIC, and had at least two extra parameters [45].

We plotted the coefficients and confidence intervals for variables in top or averaged models. We considered variables with confidence intervals that did not cross zero to be significant. To further visualize the relationships between a variable and the chance of a site being selected, we used the amt package in R [46] to plot the relative selection strength (RSS) for different values of each variable as compared to the mean value, for top models (not averaged models). The RSS provides an interpretation of the average change in probability of site selection across differing values of the covariate of interest, while all other covariates are set to fixed values, an appropriate approach for “used-available” designs [47].

## Results

### Tagging success rate and GPS locations

Of the 50 Golden-crowned Sparrows tagged across two winters, 29 were recaptured the following season, for a return rate of 59% (after excluding one bird that was recaptured the following day after deployment without a tag). The 29 recaptures included 11 of 17 deployments from Point Reyes and 18 of 32 from UC Davis (10 of 18 from the first year and 8 of 15 from the second year). Of these recaptures, three birds returned with no tag (all from Point Reyes). Of the 26 recovered tags, four had malfunctioned, resulting in 22 tags retrieved with working data. This included three of the seven birds deployed with tags twice (in two consecutive years), for a total of 19 unique individual birds with migration data. We successfully recaptured all birds with GPS tags that we resighted. The observed return rate for tagged birds was remarkably similar to that of control birds. We recaptured or resighted 28 of 46 control birds (60%): 5 of 16 from Point Reyes; 12 of 15 from UC Davis in the first year; and 11 of 15 from UC Davis in the second year.

After filtering and classifying migration locations, we had 246 spring migration locations and 295 fall migration locations (Fig. 1). After filtering out locations with unavailable environmental data, we had a total of 233 spring migration and 287 fall migration locations used in analyses. With 20 random points matched to each presence location, this resulted in 4660 spring migration random locations and 5740 fall migration random locations used in the analyses. Per individual, the number of migration locations in spring ranged from 4 to 16 (mean ± SD = 10.6 ± 3.5, n = 22 tracks) and in fall ranged from 2 to 27 (mean ± SD = 15.9 ± 6.5, n = 18 tracks).

### General description of migration

Birds from both the inland and coastal winter sites traveled along mostly coastal routes to breeding grounds predominantly in Alaska (n = 18 birds, 20 tags), or less frequently in British Columbia (n = 1 bird, 2 tags). The mean distance of migration locations from the nearest coastline was the same for both spring and fall locations (spring mean 47.4 km ± 66.8 km; fall mean 47.4 km ± 47.5 km). Locations ranged from 0.01- 409.7 km from the nearest coastline in spring, and 0.02–374.5 km in fall. The mean distance of stopover locations from the nearest coastline during migration was also very similar between the two tagging sites (Point Reyes mean = 51.4 km and UC Davis mean = 46.3 km). The mean elevation of fall migration locations (628.7 m ± 387.4 m, range = 0–1913.5 m, n = 287) was on average approximately 200 m higher than spring migration locations (417.0 m ± 354.4 km, range = 0–1905.0 m, n = 229).

The mean across birds for total distances traveled was very similar between spring (3460 km, range = 1750–4239 km, n = 22) and fall (3503 km, range = 1738–4299 km, n = 18). The duration of migration has some uncertainty, but on average spring migration duration (mean 29 days, range 17–42 days) was about a week (7.8 days) shorter than fall migration duration (36.8 days, range 15–52 days). The uncertainty in these estimates ranged from 4 to 12 days for spring migration and 2–15 days for fall migration (Table 2). The calculated travel rate (distance / duration) was 120.9 km/day in spring and 100.2 km/day in fall.

**Table 2:** Details of Golden-crowned Sparrow migration from 22 GPS tags

Bird	Year	Total days tracked	Filtered migration data points (spring/fall)	Calculated migration distance (km; spring/fall)	Minimum migration duration (days; spring/fall)	Uncertainty in duration (days; spring/fall)	Travel rate (km per day; spring/fall)	Number of longer stopovers recorded (spring/fall)
*Point Reyes*								
49209	2019	72	12/–	3674.7/–	31/–	4/–	118.5/–	1/–
49218	2019	175	13/20	4239.2/4299.3	27/41	4/9	157/104.9	0/5
49222	2019	187	13/13	3985.6/3978	38/35	11/8	104.9/113.7	1/4
49201	2019	191	16/19	4152.1/3781.6	42/52	11/11	98.9/72.7	2/4
49200	2019	63	8/–	3354.9/–	25/–	4/–	134.2/–	1/–
	Mean Point Reyes	137.6	12.4/17.3	3881.3/4019.6	32.6/42.7		122.7/97.1	1/4.3
*UC Davis*								
49206	2019	173	13/17	3446.4/3467	33/37	6/13	104.4/93.7	2/3
49771	2020	60	13/–	3430.4/–	27/–	4/–	127.1/–	1/–
49775	2020	189	9/18	3826.2/4004.8	37/42	4/15	103.4/95.4	0/2
49191	2019	191	9/16	3387.3/3617.5	29/31	> 12/2	116.8/116.7	2/4
49192	2019	163	12/10	3510.8/3474.5	27/–	4/–	130/–	2/2
49194	2019	191	13/26	3937.9/3883.9	31/–	> 2/–	127/–	1/5
49205	2019	37	10/–	3663.7/–	31/–	> 2/–	118.2/–	1/–
49217	2019	191	16/22	3976.9/3979.7	35/–	> 2/–	113.6/–	1/5
49777	2020	187	8/14	3791.3/3970	31/–	4/–	122.3/–	1/3
49780	2020	199	14/17	3869.2/3893.5	29/45	4/4	133.4/86.5	2/2
49870	2020	185	7/14	3433.2/3567.9	21/–	6/–	163.5/–	1/2
49189-a	2019	191	11/16	3158.2/3357.5	25/33	6/11	126.3/101.7	1/4
49776-a	2020	195	6/23	3127.7/3495.6	21/–	12/–	148.9/–	1/5
49195-g	2019	191	16/28	3351.9/3444.1	33/–	> 2/–	101.6/–	3/4
49770-g	2020	189	11/15	3288.4/3349.2	27/–	> 4/–	121.8/–	2/4
49202-b	2019	191	8/5	1769.4/1747	17/15	4/6	104.1/116.5	1/2
49778-b	2020	163	8/2	1750.1/1738.1	21/–	4/–	83.3/–	3/1
	Mean UC Davis	169.8	10.8/16.2	3336.4/3399.3	27.9/33.8		120.3/101.7	1.5/3.2
	Overall mean	162.5	11.2/16.4	3460.2/3502.7	29.0/36.8		120.9/100.2	1.4/3.4

Tags were deployed and recovered with data at wintering sites in northern California, and the table includes estimated distance and duration of spring and fall migration 2019–2020. Total days tracked includes the entire tracking time the tag recorded from deployment to when the tag stopped collecting points. Fall migration duration was not determined for tags that stopped collecting data during fall migration. Uncertainty is the sum of the uncertainty at both ends of migration (see “Methods” section); if information was missing for this calculation, we indicated that our estimates are potentially underestimates with “>”. No data is indicated by “–”. Bird “b” has a much lower migration distance as this bird ended its migration in British Columbia, Canada while all the other birds traveled to Alaska, USA

Four tags stopped collecting locations after arrival at the breeding areas and an additional nine tags stopped collecting locations during the return fall migration; therefore, only 9 of 22 tags recorded data for complete migrations in both seasons. Comparing spring and fall routes for the same individuals with at least partial fall migrations recorded, we found significantly more long stopovers during fall (n = 61 stopovers from 18 tags/15 birds) compared to spring migration (n = 30 across all 22 tags; t(17) = 4.8, p < 0.001; Table 2), despite fewer tags recording data throughout the entire fall migration. On average across tags, we found an increase of 1.9 long stopovers in fall compared to spring. For both fall and spring migration, both long and short stopovers were more often in shrubland than needleleaf forest or grassland (Table 3). Stopovers in needleleaf forest during both seasons were more often short than long. The proportion of locations in grassland in spring was similar for long and short stopovers, but in fall, stopovers in grassland were more often long.

**Table 3:** Number (and proportion, in parentheses) of locations that occurred in shrubland, needleleaf forest and grassland by stopover type (long or short) and by season (fall or spring) for migrating Golden-crowned Sparrows GPS-tagged at wintering grounds in California

Season	Stopover type	Total locations	Shrubland	Needleleaf forest	Grassland
Fall	Long	178	85 (0.48)	28 (0.16)	31 (0.17)
	Short	109	43 (0.39)	36 (0.33)	4 (0.04)
Spring	Long	75	36 (0.48)	11 (0.15)	4 (0.05)
	Short	158	61 (0.39)	44 (0.28)	8 (0.05)

Due to the limited frequency of recorded locations, we were not able to determine if Golden-crowned Sparrows showed site fidelity between the spring and fall seasons to stopover areas on longer stopovers. However, we did find consistency in site use between spring and fall migration for two birds at sites in Northern California: this included a female (tag 49206) that used the same stopover area (within 1 km) during one spring migration (5 GPS locations from 4/25/2019 to 5/3/2019, representing at least 9 days) as in its subsequent fall migration (6 GPS locations from 9/22/2019 to 10/2/2019 representing at least 12 days); and a male (tag 49194) that used the same stopover area (within ~ 10 km) in one spring migration (2 GPS locations from 4/19/2019 to 4/23/2019 representing at least 5 days) as its subsequent fall migration (11 GPS locations from 10/4/2019 to 10/26/2019, representing at least 23 days). Additionally, we found that one male (tag 49217) had a long stopover (at least 17 days) in the month of October within 28 km of the final wintering destination.

### Stopover analysis

**Fig. 2:**
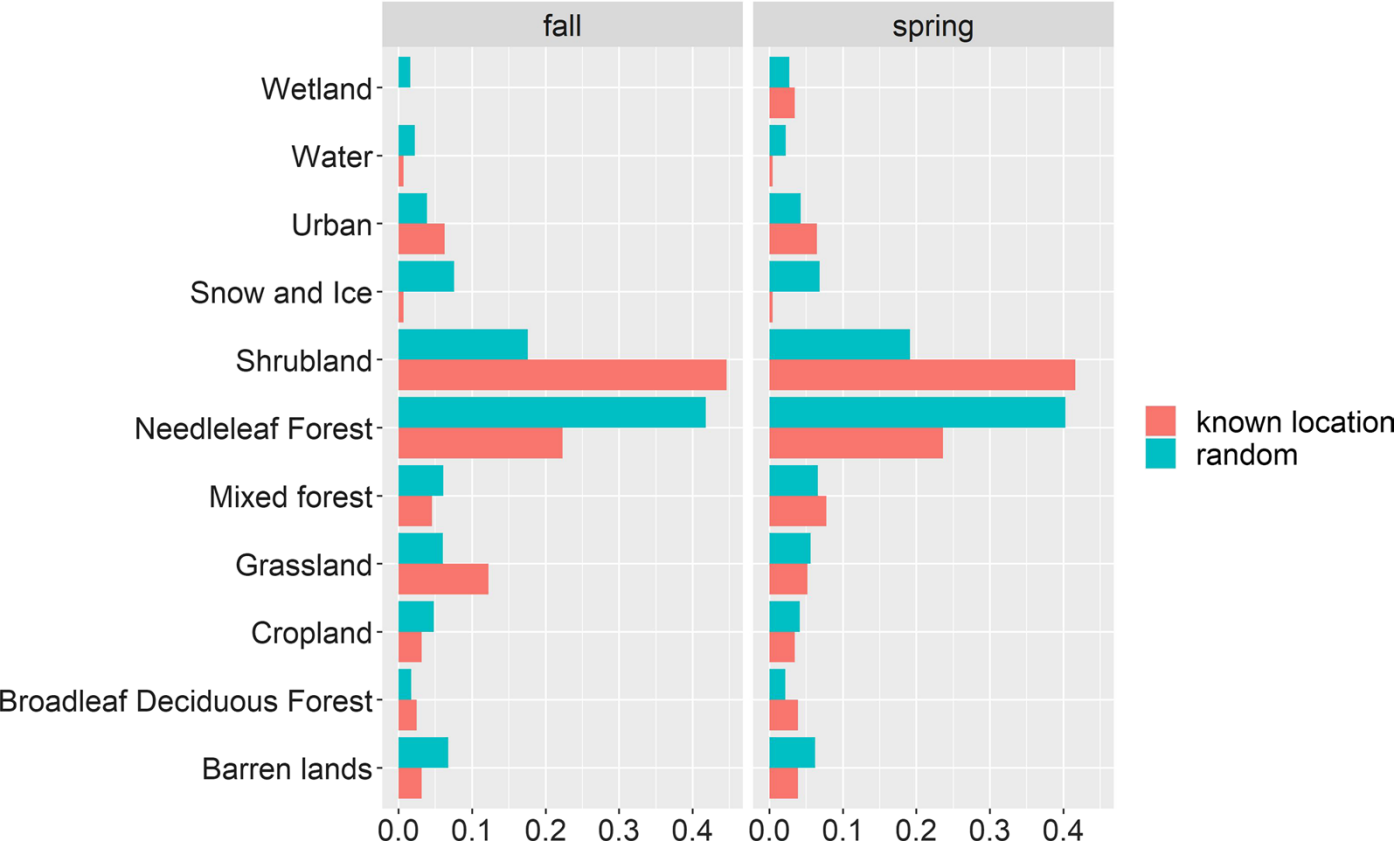
Proportion of known stopover and random points in different land cover types. Locations are shown for fall and spring migration analysis for Golden-crowned Sparrows migrating to/from wintering locations in northern California, 2019–2020. Land cover types are abbreviated: Shrubland = Temperate or sub-polar shrubland, Needleleaf Forest = Temperate or sub-polar needleleaf forest, Grassland-Lichen-Moss = Sub-polar or polar grassland-lichen-moss, Grassland = Temperate or sub-polar grassland, and Broadleaf Deciduous Forest = Temperate or sub-polar broadleaf deciduous forest. There were only 5 random points in grassland-lichen-moss (4 in fall and 1 in spring) meaning the proportions were very close to zero for this land cover type, so it is not presented. There were no presence locations in wetland in fall

NDVI, daily precipitation, and temperature values were relatively similar across known stopover locations as compared to random locations, and random locations covered a wider range of values (Table 4). Twelve land cover classes were represented at both known stopover and random locations, with needleleaf forest (for random locations) and shrubland (for known stopover locations) the two most common (Fig. 2).

**Table 4:** Comparison of environmental data at known stopover and random locations

	NDVI (p)	NDVI (r)	Precip (p)	Precip (r)	Daily max temp (p)	Daily max temp (r)	Daily min temp (p)	Daily min temp (r)
*Spring*								
Min	− 0.10	− 0.20	0.00	0.00	4.91	− 9.10	− 6.79	− 19.55
Max	0.91	0.94	31.70	45.70	31.22	33.54	16.15	17.92
Mean	0.61	0.55	1.61	1.65	17.74	16.63	5.22	4.60
SD	0.23	0.32	3.88	4.06	5.62	6.44	4.05	4.42
*Fall*								
Min	− 0.09	− 0.19	0.00	0.00	1.24	− 15.68		
Max	0.93	0.99	79.85	103.78	35.93	37.22		
Mean	0.64	0.61	5.24	5.29	17.10	16.64		
SD	0.19	0.28	10.00	10.16	6.50	6.61		

The minimum (min), maximum (max), mean and standard deviation (SD) of environmental data values extracted across all presence (p) and random (r) locations. Environmental values include NDVI, daily precipitation (mm; Precip), maximum daily temperature (°C; Max Temp) and minimum daily temperature (°C; Min Temp) at known stopover (p, n = 233 for fall and n = 287 for spring) and random (r, n = 4660 for spring and n = 5740 for fall) locations for spring and fall migration

**Fig. 3:**
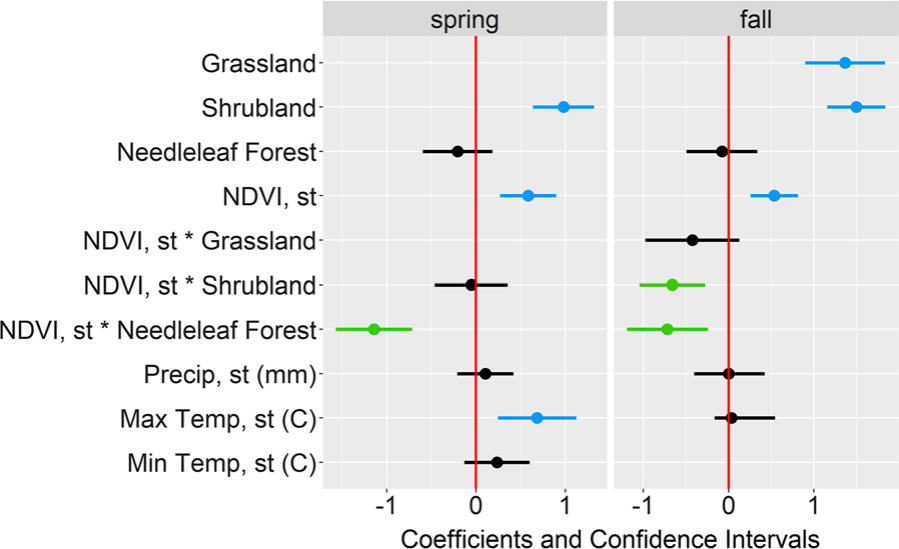
Coefficients and confidence intervals from conditional logistic regression migration habitat selection models for Golden-crowned Sparrows. The left panel is for the spring migration top model and the right is for the model averaged results for fall. Golden-crowned Sparrows wintered in northern California and were tracked in 2019–2020. Dots represent coefficient values and lines represent confidence intervals. Positive relationships are shown in blue and negative are shown in green. Variables with “st” indicate they were standardized. C = Celsius

**Fig. 4:**
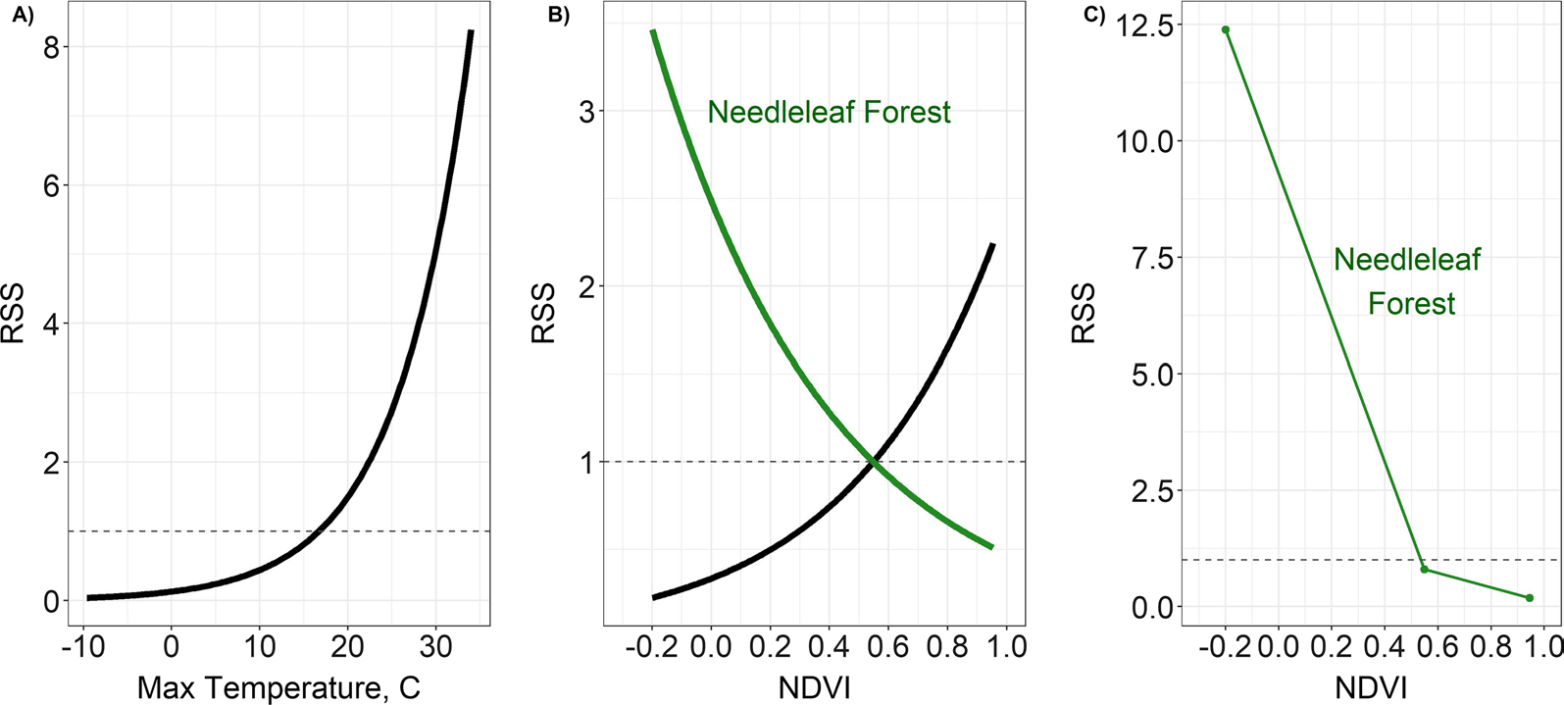
The relative selection strength (RSS) results for habitat selection along spring migration in 2019–2020 for Golden-crowned Sparrows wintering in northern California. **A** The RSS for sites depending on the daily maximum temperature (Max Temperature), **B** The RSS for NDVI, and **C** the RSS for needleleaf forest habitat at different NDVI values. In panel B, the black line shows the RSS for NDVI in the absence of the habitat types in the model (needleleaf forest and shrubland). The green line shows RSS for NDVI when needleleaf forest is present. Panel C shows the selection strength for needleleaf forest when other habitat types in the model are absent (shrubland) and at minimum, mean, and maximum NDVI values to demonstrate the interaction of needleleaf forest and NDVI. These graphs compare the likelihood of selecting a site compared to another site set at the mean value of the variable (or for panel C, to absence of the habitat type). The logarithmic value of the RSS is zero when the two locations being compared are identical (i.e. RSS = 1); therefore, the dashed line represents “no selection” as it intersects with the mean value (or zero for panel C)

The global model was the top model for spring migration (Table 5), during which birds selected for warmer temperatures, higher NDVI values and the presence of shrubland (Fig. 3). Specifically, the relative selection strength (RSS) for maximum daily temperature shows that compared to the mean maximum temperature (16.7 ℃) across all random and presence points, birds are five times more likely to select for sites with a daily maximum temperature of 30℃ (Fig. 4A). The RSS for NDVI during spring migration depended on the habitat type, as indicated by the significant interaction in the model. For example, compared to a site with the mean NDVI value (0.55 across all presence and random points), birds are twice as likely to select a site with ~ 0.9 NDVI (Fig. 4B, black line), when shrubland and needleleaf forest are absent. However, this relationship changed when needleleaf forest habitat was present, such that birds were more likely to select for sites with lower NDVI in needleleaf forest habitat (Fig. 4B, green line). When looking at the selection strength of needleleaf forest, birds were more likely to select needleleaf forest at minimum NDVI values than maximum NDVI values (Fig. 4C). The interaction between shrubland and NDVI in the spring was not significant (Fig. 3).

**Table 5:** Models compared for stopover selection along spring and fall migration in 2019–2020 by Golden-crowned Sparrows

Models	Δ AIC_c_	weight
*Spring migration*		
Shrub + NeedFor + NDVI + (NDVI * Shrub) + (NDVI * NeedFor) + Prcp + Tmax + Tmin	0	0.995
Shrub + NeedFor + NDVI + (NDVI * Shrub) + (NDVI * NeedFor)	10.52	0.005
Shrub + NeedFor + NDVI + Prcp + Tmax + Tmin	24.46	0
Shrub + NeedFor + NDVI	38.94	0
Shrub + NeedFor	64.14	0
Prcp + Tmax + Tmin	96.69	0
NDVI	114.85	0
*Fall migration*		
Grass + Shrub + NeedFor + NDVI + (NDVI * Grass) + (NDVI * Shrub) + (NDVI * NeedFor)	0.0	0.809
Grass + Shrub + NeedFor + NDVI + (NDVI * Grass) + (NDVI * Shrub) + (NDVI * NeedFor) + Prcp + Tmax	3.09	0.172
Grass + Shrub + NeedFor + NDVI	8.87	0.010
Grass + Shrub + NeedFor + NDVI + Prcp + Tmax	9.72	0.006
Grass + Shrub + NeedFor	11.34	0.003
Prcp + Tmax	154.36	0
NDVI	154.84	0

Models compared include variables: Shrub = shrubland, NeedFor = needleleaf forest, NDVI, Prcp = daily precipitation, Tmax = daily maximum temperature, and Tmin = daily minimum temperature. Δ AIC_c_ = delta AIC values and weight = model weights. The AIC for the top model in spring was 1287.2 and for fall it was 1588.9

**Fig. 5:**
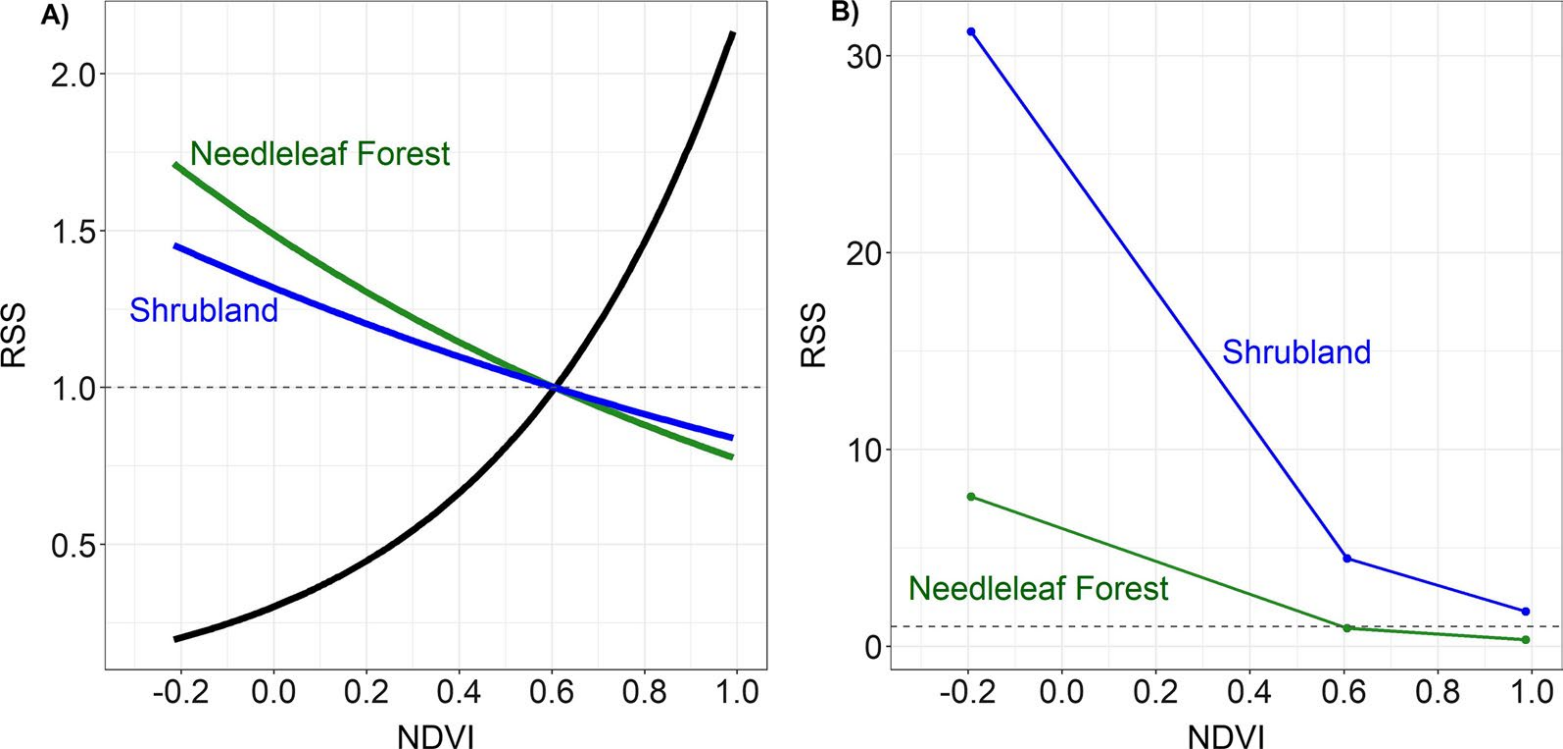
The relative selection strength (RSS) results for habitat selection along fall migration in 2019–2020 for Golden-crowned Sparrows wintering in northern California. **A** The RSS for NDVI and **B** the RSS for habitat types across different NDVI values. In panel A, the black line shows the RSS for NDVI in the absence of the land cover types in the model (needleleaf forest, shrubland and grassland). The green line shows the RSS for NDVI when needleleaf forest is present and the blue line shows the RSS for NDVI when shrubland is present. Panel B shows the selection strength for needleleaf forest and shrubland when other habitat types in the model are absent for minimum, mean, and maximum NDVI values to further demonstrate the interaction of habitat types and NDVI. These graphs compare the likelihood of selecting a site compared to another site set at the mean value of the variable (panel A), or between presence and absence of the habitat types (panel B; specifically, the comparison is between presence of the habitat type to absence of all habitat types in the model). The logarithmic value of the RSS is zero when the two locations being compared are identical (i.e. RSS = 1); therefore, the dashed line represents “no selection” as it intersects with the mean value (panel A) or absence of the habitat types (panel B)

There were two models for fall migration that were < 4.0 Δ AIC_c_, the global model and a simpler version of the global model without precipitation and maximum temperature (Table 5). The averaged model showed that NDVI, the presence of shrubland, the presence of grassland, the interaction between NDVI and needleleaf forest, and the interaction between NDVI and shrubland were all significant (Fig. 3). On fall migration, birds selected for higher NDVI values and the presence of shrubland and grassland. Similar to spring migration, the relative selection strength for NDVI during fall migration depended on the habitat type, as indicated by the significant interactions in the model. RSS plots show that birds were more likely to select for higher NDVI when habitat types included in the model were absent (i.e. no needleleaf forest, shrubland or grassland); however, this relationship was negative when either shrubland or needleleaf forest were present. That is, when choosing between sites with certain habitat types (needleleaf forest or shrubland), birds were less likely to choose sites with higher NDVI values (Fig. 5A). Similar to spring, when looking at the selection strength of needleleaf forest, birds in the fall were more likely to select needleleaf forest (when other habitat types in the model were absent) at minimum NDVI values than maximum NDVI values (Fig. 5B). The same pattern was true for shrubland, and the selection strength for shrubland was always higher than for needleleaf forest at all NDVI values (Fig. 5B).

**Fig. 6:**
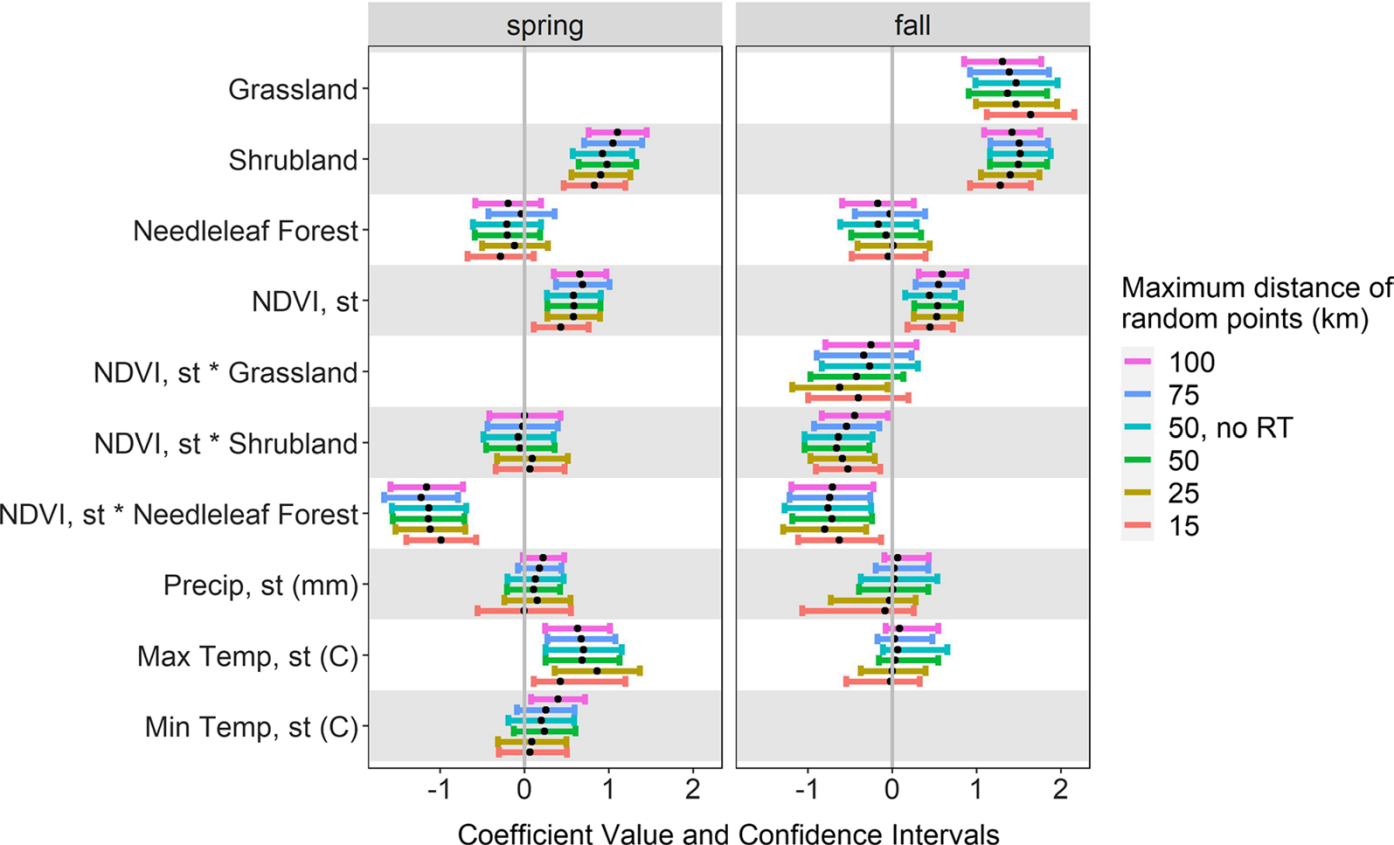
Results of the sensitivity analysis for habitat selection at stopovers sites by Golden-crowned Sparrows. Birds wintered in northern California and migrations were tracked in 2019–2020. The sensitivity analysis involved the creation of random points in different definitions of available habitat, including random points up to 15, 25, 75 and 100 km from each matched presence point. The 50 km results are reported as the main results and are shown for reference. The 50 km results with repeat tracks from three birds removed is labeled “50, no RT”. Variables with “st” indicate they were standardized. Max = maximum, Min = minimum, Temp = temperature, C = Celsius

The sensitivity analysis indicated consistency in our results at different scales of available habitat. That is, coefficient estimates and confidence intervals were similar for both spring and fall migration as compared to the main analysis. The only exceptions were: (1) for spring migration, selection for sites with a higher minimum temperature was significant at the largest scale (100 km), but not significant at smaller scales; and (2) for fall migration, the interaction between NDVI and grassland was significantly negative at 25 km while at other scales this interaction was not significant (Fig. 6). Additionally, the analysis where one track was removed from each of the three birds tracked twice showed similar results as the main analysis (Fig. 6).

## Discussion

We found that land cover type is important in migration habitat selection for Golden-crowned Sparrows migrating between California and, predominantly, coastal Alaska. Specifically, shrubland areas were preferentially selected during both spring and fall migration. This is consistent with earlier work identifying shrubland as important at breeding and wintering grounds [12], and confirms that shrubland is important for this species during all stages of the annual cycle on a macrohabitat scale. We also found that grassland was important in fall migration.

We expected Golden-crowned Sparrows to avoid densely-forested habitat; however, while birds did not select for needleleaf forest, they also did not completely avoid it. This may be due to the prevalence of such forests along their migratory routes, as corroborated by the relatively high proportion of random locations in this type of forest. However, on the breeding grounds this species has been observed in scattered conifers near the tree line in Alaska [12] indicating they don’t avoid conifers completely, and we found stopovers in this habitat type were more often short. Land cover was classified by the NALCMS as temperate or sub-polar needleleaf forest when the forests in each grid cell were taller than three meters, covered more than 20% of total vegetation, and at least 75% of the canopy cover contained needle-leaved species. Therefore, it is possible for a grid cell classified as needleleaf forest to range in needleleaf coverage from 20 to 100%, and this range may explain the negative interaction between NDVI and needleleaf forest that we found in both migration seasons: the higher the NDVI and potentially denser the vegetation (higher greenness), the less likely birds were to use needleleaf forest. The NDVI values and the negative interaction we found could also be influenced by the need to improve NDVI estimates from satellite imagery in evergreen forests with improved characterization of canopy structure and leaf biochemistry [48].

Land cover was classified as temperature or sub-polar shrubland by the NALCMS when (typically) more than 20% of the vegetation was dominated by woody perennials with persistent woody stems less than three meters in height. We found a similar negative interaction with NDVI and shrubland in fall migration: as NDVI values increased, birds were less likely to choose that habitat. Lower NDVI is a result of less vegetation greenness, so could be due to the shrubland being more open (i.e. closer to 20% than 100% of woody perennial coverage), or potentially less leafy (i.e. if plants have dropped their leaves during fall migration). Given the prevalence of evergreen to semi-deciduous shrub species in the west, this suggests that within the preferred shrubland habitat during migration, there is also a preference for more open shrublands. Similar to our results during migration, Golden-crowned Sparrows on the breeding grounds were found to be more abundant in areas with lower shrub heights, and predicted to be at higher abundances with increasing shrub densities but only to a certain threshold (~ 60 shrubs per 100 m^2^) at which point abundances plateaued [49]. However, this interaction between habitat type and NDVI may also represent a trade-off between preferred habitat types and productivity on the landscape. Previous work on terrestrial migrating bird species using eBird data showed that NDVI was able to predict species richness to some degree, however geography (distance to the coast) played a more important role, and species richness even declined slightly with higher NDVI values [50]. Additionally, studies on long-distance migratory birds found most species tracked climate and land cover more than vegetation productivity [51]. However, other studies on migratory birds have found positive relationships between species richness or presence and some measure of vegetation productivity [18, 52, 53]. The positive relationships to NDVI and shrubland we found, along with the negative interaction between them, suggests that when shrubland is present, high NDVI values are selected for less, and/or, when NDVI values are high, shrubland is selected for less strongly. With higher NDVI values available, and therefore presumably more resources for refueling, specific habitat types may become less important during migration. With lower NDVI values and presumably less resources for refueling, birds may focus on their preferred habitat types. Trade-offs may also exist in another way, such that the costs of the distance traveled offset better access to resources [54]. The negative interaction between NDVI and shrubland was only present in fall migration, so if trade-offs do exist, they are presumably less important during spring migration (and not present for grasslands in the fall).

For Golden-crowned Sparrows and other short-bout migrants that need to refuel during migration, habitat selection during migration is likely related to food resources. Golden-crowned Sparrows are omnivores, but other than feeding insects to their young, it is thought they only eat insects opportunistically [55]; thus any association with NDVI is more likely related to the plant resources themselves, not productivity cascading up into insect availability. Stomach contents from overwintering and migrating birds in California included mostly buds, flowers, grain and seed (97–99% plant material), and very little insect matter [55]. Other than nestling fecal sacs, only plant material has been recorded in summer diets, although summer diet is not well documented [12]. In fall in California, birds were primarily consuming a seed diet beginning in October, and gradually changed to a bud and flower diet from December to April [55]. If this trend persists to this day and into their migratory periods, this could indicate that Golden-crowned Sparrows focus on flowers and buds during spring migration and grains and seeds during fall migration, possibly switching their habitat preference accordingly. Although quantitative evidence for this diet switch is lacking, they have been observed to feed extensively on flowers on their wintering grounds in spring when flowers become prevalent in California (D. Humple, pers. obs.).

Analyses from 57 migratory landbird species from western North America found that many species take a loop approach, tracking ecological productivity by selecting lower-elevation routes in spring and then taking higher elevation routes in fall that tracked productivity less but potentially minimized the distance traveled [18]. While we did not find differences between spring and fall migration routes in the total distance traveled or mean distance from the coast that would indicate a loop migration for Golden-crowned Sparrows, we did find that spring migration locations were around 200 m on average lower in elevation than fall migration locations. We also found a positive relationship to NDVI for both seasons, suggesting that Golden-crowned Sparrows are tracking ecological productivity in both migration seasons. Our sample of birds traveled primarily to Alaska (and one to British Columbia), while previous work has shown a regional difference in migratory routes and breeding areas for birds wintering in two different regions of California [13]. Future research on populations that breed in Canada could reveal if these findings are consistent across populations.

While climate variables were in our top models, we found strong support for an effect during spring, though this was driven primarily by maximum temperature, and only weak support for an effect during fall (Δ AIC_c_ > 2 and low weight). This is in line with previous studies in western North America that did not find that species’ migration routes tightly tracked precipitation patterns (summarized in [18]). However, we found that Golden-crowned Sparrows selected sites with a higher daily maximum temperature during spring migration. In one experimental study of a closely-related species, White-throated Sparrows exposed to cold temperatures (− 20 ℃) needed 83% more food to maintain body mass than birds held at 21 ℃. It takes time for birds to acclimate to colder temperatures as they need to increase their gut size to accommodate a higher feeding rate, with larger digestive organs during migration likely incurring a higher energic cost [56]. Therefore, migrating birds would benefit from avoiding rapid decreases in temperature. It has also been well documented that many migratory species advance their spring migration timing in response to warmer temperatures [57, 58], including in western North America [59]. While it is unclear if selecting for warmer sites during migration translates to flexibility with climate change, this species has shown flexibility in migration phenology demonstrated by long-term banding data at a nearby site in Point Reyes National Seashore that shows an earlier arrival time to wintering grounds of about 8 days during a 36 year period (1979–2015) [60].

Golden-crowned Sparrows have shown high site fidelity to wintering areas ([12–14], Point Blue unpubl. data) residing in small areas (~ 7 ha) [61]. However, passerine site fidelity to stopover sites is less well understood [62]. Previous banding studies at a stopover site for White-crowned Sparrows, a closely related species, showed no recaptures across years for more than 6000 banded birds [63]. With our temporal resolution (every 2 days) of data collection during migration, we were able to detect that two individuals used the same stopover area (one to within 1 km) in both their spring and fall migration. The frequency with which individuals use the same stopover areas every year is still poorly known. Repeat use of a stopover area across years, and the specificity to an exact site or territory within that area, might depend on the reliability of resources at those sites or scarcities at other sites. Future studies determining the level of site fidelity to stopover areas during migration could help us understand variability in migratory routes and give clues on the flexibility of migrants to changing conditions. Multi-year fine-scale tracking studies of individuals would be helpful to determine the frequency and scale of stopover site fidelity.

Birds are thought to migrate in a more “relaxed” fashion on fall migration than spring migration, as they are under pressure to arrive early to breeding grounds to establish good territories and begin nesting earlier, which increases reproductive success [64]. While we could not determine exact migration durations because we were not taking daily readings, we did find evidence to suggest that the total time on migration in fall was about a week longer than on spring migration, and that during fall migration birds stopped more often for long periods (defined as at least 3 days). Previous studies using light-level geolocators on California-wintering Golden-crowned Sparrows found that migration was nearly twice as fast during spring than fall (n = 4 coastal-wintering birds; [14]), and spring migration rate (distance/duration) was faster for coastal-wintering birds than inland-wintering birds (n = 9 coastal birds; n = 8 inland birds; [13]). Light-level geolocator tags collect data on a daily basis but have larger spatial errors than GPS tags [65]. With limited battery life for small GPS tags, there is a tradeoff between frequency of points and the length of time a bird is tracked. Therefore, while the tags in this study were more spatially accurate than previous studies based on light-level tags, we did not collect daily migration data and this introduced some uncertainty in our migration duration estimates. Nevertheless, estimates of duration timing from this and previous studies strongly suggest that for Golden-crowned Sparrows, fall migration is longer than spring migration. Based on the greater prevalence of long stopovers in fall, this study also supports that birds are traveling more slowly in fall than spring.

An important consideration in the length of stopover time is the physiological transition between a migratory phase and refueling phase, as birds may change their gut structure to prepare for migration and would need time to physiologically switch between the two phases during stopovers [11]. Studies of passerines at stopover sites have shown a slow recovery in body condition for the first 1 or 2 days after arrival even with abundant food available, and then a rapid recovery following this [56]. Digestive constraints can slow the pace of migration as changes in gut size and digestive enzymes are needed to recover from fasting or accommodate switches in diet (as may occur during migration) and this can take days [56]. Further, poor body condition upon arrival has been linked to longer stopovers, presumably because the bird needs more time to gain reserves to continue migration [11]. Therefore, it is plausible that a greater frequency of longer stopovers in fall could be due to the food resources encountered along the route, such as less consistency in food type, a lower nutritional quality, or less abundance as compared to spring migration. Another factor that could affect nutritional needs and time spent refueling could be recovery from post-breeding molt and/or poor body condition after breeding, though this did not affect arrival time to wintering grounds for wood thrushes (*Hylocichla mustelina*; [66]). However, the shorter migration times in spring may not be solely related to resource availability, but also to the selective benefit to arrive early at breeding grounds, resulting in differences in flight speeds, foraging rates and fuel deposition [67].

Migration can have direct and indirect effects on survival and indirect effects on reproduction. For example, 85% of apparent annual mortality of Black-throated Blue Warblers (*Setophaga caerulescens*) occurred during migration [4]. Virtually nothing is known about the factors that regulate populations of Golden-crowned Sparrows, although nestling mortality (i.e. breeding productivity) may be an important factor during the breeding season [12]. There are no estimates of survival probability during migration for Golden-crowned Sparrows, as is the case for many passerines [5]. Understanding migration routes and stopover choices is ever more urgent as bird species are showing declines nation-wide [2]. Although the IUCN Red List categorizes Golden-crowned Sparrow as “Least Concern” with stable or possibly increasing populations, it is rarely monitored with the Breeding Bird Survey (BBS) as the species generally nests away from BBS routes. According to long-term Christmas Bird Count (CBC) data, Golden-crowned Sparrows are generally increasing across much of their wintering range, but showing declines in the more southern portions (e.g., Coastal California and Sonoran and Mojave Deserts Bird Conservation Regions; [68]). The declines in southern areas are consistent with a possible wintering range shift to the north due to climate change, although could also be due to other factors like habitat loss or degradation.

Temperature and precipitation are expected to change over the next twenty years throughout the range of the Golden-crowned Sparrow [69]. These changes may have direct impacts on migration phenology through changing temperature and wind conditions at stopover grounds [57, 70] or indirect effects due to habitat shifts. Of the habitat types Golden-crowned Sparrows selected during migration, the cover of grassland is expected to increase in North America under future climate change scenarios and have a longer growing season [71]. Additionally, non-native grasses are expected to increase as they invade shrublands in western North America after fires [72]. Predictions for shrubland are more mixed, with shrubland expected to replace some tundra and conifer habitats in North American national parks [73], but also show varying degrees of increase or decrease throughout the interior west [74]. For areas near the coast specifically, biome change is expected to be more limited [75] and changes in anthropogenic land use are a larger threat than climate change for many species of California sage scrub [76]. Based on these mixed predictions for changes in habitat type across their range, it is difficult to predict how Golden-crowned Sparrows would be affected. An increase in preferred land cover (grassland in the fall and shrubland both seasons) might seem beneficial for migration, but it is less clear how Golden-crowned Sparrows would react to synergistic effects of habitat shifts, seasonality, fire, and habitat loss from human development. At a breeding area in Alaska for example, Golden-crowned Sparrows show evidence of shifting to higher elevations, but there is limited potential for shrub habitat to occur above 1200 m in those areas [77].

## Conclusions

Our results show that land cover class and NDVI are important aspects of habitat selection for migrating Golden-crowned Sparrows as birds select for shrubland (both seasons) and grassland (during fall migration), and generally prefer productive areas (areas with higher NDVI). The relationship to NDVI varied by habitat, potentially reflecting a preference for more open habitats, or representing a trade-off in selection between habitat type and productivity. By demonstrating that shrubland is important in this study, we confirm that this land cover class is important in all life stages for this species. Data from GPS tags provided us with information on small songbird migration that was previously unknown and not often recorded for other species of similar size and migratory behavior. For example, we found repeated use of stopover areas for individual birds between their spring and fall migration. We also found birds had a higher frequency of long stopovers during fall migration. While conservation efforts often focus on rare species, common species have disproportionate effects on ecosystems through their relative abundance [78]. A decline in these species, while not necessarily bringing them close to extinction, could have larger effects on ecosystems than losing more rare species. Using miniaturized GPS, this study provides new insight into habitat selection along migration routes for one such common temperate-zone migrating songbird, contributing to a better understanding of full annual cycle models, and informing conservation efforts.

## Data Availability

The datasets generated and/or analyzed during the current study are available: via Movebank (movebank.org) for migration locations, https://doi.org/10.5067/MODIS/MOD13A2.006 for NDVI, https://doi.org/10.3334/ORNLDAAC/1840 for Daymet (climate variables), http://www.cec.org/nalcms for land cover classes and https://ftp.maps.canada.ca/pub/nrcan_rncan/elevation/canada3d/can3d30.zip for elevation in Canada.

## Abbreviations

NDVI: Normalized Difference Vegetation Index
GPS: Global Positioning System
HDOP: Horizontal Dilution of Precision
RSS: Relative Selection Strength
NALCMS: North America Land Change Monitoring System
